# *MECP2* Duplication Syndrome: Evidence of Enhanced Oxidative Stress. A Comparison with Rett Syndrome

**DOI:** 10.1371/journal.pone.0150101

**Published:** 2016-03-01

**Authors:** Cinzia Signorini, Claudio De Felice, Silvia Leoncini, Rikke S. Møller, Gloria Zollo, Sabrina Buoni, Alessio Cortelazzo, Roberto Guerranti, Thierry Durand, Lucia Ciccoli, Maurizio D’Esposito, Kirstine Ravn, Joussef Hayek

**Affiliations:** 1 Department of Molecular and Developmental Medicine, University of Siena, Siena, Italy; 2 Neonatal Intensive Care Unit, Azienda Ospedaliera Universitaria Senese, Siena, Italy; 3 Child Neuropsychiatry Unit, Azienda Ospedaliera Universitaria Senese, Siena, Italy; 4 Danish Epilepsy Centre, Dianalund, Denmark; 5 Institute for Regional Health Services, University of Southern Denmark, Odense, Denmark; 6 Department of Medical Biotechnologies,University of Siena, Siena, Italy; 7 Institut des Biomolécules Max Mousseron (IBMM), UMR 5247-CNRS-UM-ENSCM, Montpellier, France; 8 Institute of Genetics and Biophysics “A. Buzzati-Traverso”, Naples, Italy; 9 IRCSS Neuromed, Pozzilli, Italy; 10 Department of Clinical Genetics, Copenhagen University Hospital Rigshospitalet, Copenhagen, Denmark; King Faisal Specialist Hospital and Research Centre, SAUDI ARABIA

## Abstract

Rett syndrome (RTT) and *MECP2* duplication syndrome (MDS) are neurodevelopmental disorders caused by alterations in the methyl-CpG binding protein 2 (*MECP2*) gene expression. A relationship between *MECP2* loss-of-function mutations and oxidative stress has been previously documented in RTT patients and murine models. To date, no data on oxidative stress have been reported for the *MECP2* gain-of-function mutations in patients with MDS. In the present work, the pro-oxidant status and oxidative fatty acid damage in MDS was investigated (subjects n = 6) and compared to RTT (subjects n = 24) and healthy condition (subjects n = 12). Patients with *MECP2* gain-of-function mutations showed increased oxidative stress marker levels (plasma non-protein bound iron, intraerythrocyte non-protein bound iron, F_2_-isoprostanes, and F_4_-neuroprostanes), as compared to healthy controls (P ≤ 0.05). Such increases were similar to those observed in RTT patients except for higher plasma F_2_-isoprostanes levels (P < 0.0196). Moreover, plasma levels of F_2_-isoprostanes were significantly correlated (P = 0.0098) with the size of the amplified region. The present work shows unique data in patients affected by MDS. For the first time *MECP2* gain-of-function mutations are indicated to be linked to an oxidative damage and related clinical symptoms overlapping with those of *MECP2* loss-of-function mutations. A finely tuned balance of *MECP2* expression appears to be critical to oxidative stress homeostasis, thus shedding light on the relevance of the redox balance in the central nervous system integrity.

## Introduction

Methylated-CpG binding protein 2 (MeCP2) is a nuclear protein encoded by the X-linked *MECP2* gene (OMIM*300005). MeCP2 can be defined as a multifunctional protein, due to its involvement in chromatin architecture, regulation of RNA splicing, and its role both as transcriptional repressor or activator [1]. *MECP2* appears to be universally expressed in all cell types with few exceptions, including microglia and rod photoreceptors [2]. Loss-of-function mutations in *MECP2* is the main cause of Rett syndrome (RTT), which is a neurodevelopmental disease with severe cognitive impairment occurring at a ratio of approximately 1:10,000 girls [3,4].

However, RTT is not the only known pathological condition related to *MECP2* mutations, as a wide series of conditions, collectively termed as *MECP2*-related disorders [5], has been reported. These disorders include asymptomatic female carriers, boys with *MECP2* mutations typically causing a RTT phenotype in girls, and rare individuals with mutations in *MECP2* showing other neurodevelopmental disorders [5]. Interestingly, intellectual disability (ID) is a common feature between RTT as well as *MECP2*-related disorders. Gain-of-function mutations in relation to *MECP2* also lead to a severe neurodevelopmental disorder, named *MECP2* duplication syndrome (MDS) or *MECP2* triplication syndrome [6–8]. The phenotypes include major cognitive and motor deficits, stunted motor development, early onset hypotonia, epilepsy, and progressive spasticity, clinical features which are overlapping with some of those seen in RTT [6,7].

Although the prevalence of MDS is unknown, it has been estimated that MDS could be responsible for 1 to 2% of all X-linked ID cases [9]. MDS is well documented in males, with 150 affected individuals reported in the literature, while it rarely occurs in females [9], as female carriers of *MECP2*/Xq28 duplications are almost always asymptomatic due to extremely skewed X-inactivation [9]. This is in contrast to females with classical RTT harboring a loss-of-function mutations of the *MECP2* [10,11].

Interestingly, a genotype-phenotype correlation in relation to the size of the Xq28 duplicated region has emerged. The minimal region of duplication that is sufficient to cause the core MDS phenotype involves the *MECP2* and *IRAK1* [12–14]. Evaluating previously data and data from mouse models, Ramocki and colleagues posit that *MECP2* is the primary dosage-sensitive gene responsible for the neurological phenotypes in the Xq28 duplications [7].

Overall, MeCP2 appears to play a key role in the brain as a regulator of synaptic and neuronal plasticity as well as an etiological role in the development of RTT and MDS [15].

Oxidative stress (OS) is a nonspecific pathological condition that has frequently been associated with neurological disorders, including several diseases linked to cognitive impairment [16]. Compelling evidence between *MECP2* loss-of-function and aberrant redox homeostasis has been shown by our and other research groups [17,18]. Specifically, a cause-effect relationship between oxidative brain damage and *Mecp2* loss-of-function has been reported by our group in several murine models of the disease [19].

Since both a critical role for *MECP2* in central nervous system integrity [20], and an involvement of OS in brain synaptic plasticity have been reported [21–23], MDS can represent a clinical model for testing the hypothesis that a finely tuned balance of *MECP2* expression is critical to the control of redox homeostasis. We hypothesize that overexpression of *MECP2* would lead to a status of enhanced OS.

## Materials and Methods

### Subjects

The study included a total of 42 subjects. Six subjects carrying an Xq28 duplication/triplication (ranging from 0.426 to 3.9 Mb) all-encompassing *MECP2* (female n = 1, males n = 5; mean age 10.7 ± 4.3 years, range: 2.5–14) were enrolled in the study. One patient (case #6) was admitted at the Danish Epilepsy Centre, Dianalund, Denmark, while five patients (cases #1–5) were followed at the Child Neuropsychiatry Unit, Azienda Ospedaliera Universitaria Senese, Siena Italy. For comparative purpose, “positive” controls (RTT patients with proven *MECP2* loss-of-function mutations; n = 24; all females; mean age 10.1 ± 3.3 years, range 3–14) were also analyzed, as a part of cohort of patients on regular follow-up at the Child Neuropsychiatry Unit, University Hospital, Siena Italy. All the RTT patients were identified with a *MECP2* mutation and clinically evaluated according to the revised diagnostic criteria by Neul et al. [24]. The patients were all diagnosed with the classical form of RTT [4]. In addition, “negative” controls were newly recruited (healthy subjects n = 12; males 4, females 8; mean age 10.3 ± 3.7 years, range 3–14). Age differences were not statistically significant (P = 0.927).

The work was carried out in accordance to the rules expressed in the Declaration of Helsinki, and written informed consent was obtained by the parents of the enrolled subjects. This study was approved by the competent institutions: Ethics Committee of Western Zealand, Denmark (for the patient admitted at the Danish Epilepsy Centre), and Ethics Committee of the Tuscan Region, Azienda Ospedaliera Universitaria Senese, Siena, Italy (for the subjects recruited at the Child Neuropsychiatry Unit).

### Microarray-comparative genomic hybridisation (array-CGH) analysis

The Xq28 duplications were identified by Array-CGH analysis using Agilent oligonucleotide array kit 44B (Human Genome CGH Microarray Kit 44B; Agilent Technologies, Santa Clara, CA), with an average resolution of about 75 kb or by a sub-megabase resolution whole genome tiling path BAC array (http://www.molgen.mpg.de/~abt_rop/molecular_cytogenetics/). The duplications were confirmed by real-time quantitative polymerase chain reaction (qPCR) [25].

### Blood sampling

Blood sampling was carried out after the overnight fast. Blood samples were collected in heparinized tubes, and centrifuged at 2,400Xg for 15 min at room temperature. The platelet poor plasma was saved, and the buffy coat was removed by aspiration. Erythrocytes were washed twice with physiological solution, suspended in Ringer solution, pH 7.4 as a 50% (vol/vol) suspension, and then used for the determination of intraerythrocyte non-protein bound iron (IE-NPBI). Plasma was used for free F_2_-isoprostanes (F_2_-IsoPs), F_4_-neuroprostanes (F_4_-NeuroPs), F_2_-dihomo-isoprostanes (F_2_-dihomo-IsoPs), and plasma non-protein bound iron (p-NPBI). For all isoprostane determinations, butylated hydroxytoluene (BHT) (90 μM) was added to plasma as an antioxidant.

### Oxidative stress marker evaluations

The examined markers included non-protein bound iron (NPBI) (pro-oxidant factor), F_2_-IsoPs (the oxidized products from arachidonic acid and markers of OS/systemic lipoperoxidation), F_4_-NeuroPs (the oxidized products from docosahexaenoic acid and markers of neuronal membrane damage/brain gray matter), and F_2_-dihomo-IsoPs (the oxidized products from adrenic acid and markers of glia membrane damage/brain white matter) [17]. OS markers were measured as previously reported. Briefly, IE-NPBI and p-NPBI were determined as a desferrioxamine-iron complex by high-performance liquid chromatography [26], whereas F_2_-IsoPs, F_4_-NeuroPs, and F_2_-dihomo-IsoPs were determined by a gas chromatography/negative ion chemical ionization tandem mass spectrometry (GC/NICI-MS/MS) analysis [26–28].

### Routine blood tests

Serum concentrations of total cholesterol, high density lipoproteins (HDL)-cholesterol, triglycerides, immunoglobulin class G, class A and class M (IgG, IgA, IgM), were performed by a fully automatic analyzer (Cobas 6000 System; Roche Diagnostics). Total cholesterol, HDL-cholesterol, and triglycerides were assayed by using an enzymatic methods [29–31]. Serum fibrinogen concentration was determined by using Thromborel Reagent on BCS XP coagulation analyzer (Siemens Healthcare) [32]. Erythrocyte Sedimentation Rate (ESR) was assayed by using an automated system [33]. Blood cells counts were assayed on Sysmex XT-2100 system [34].

### Data analysis

All variables were tested for normal distribution (D’Agostino-Pearson test). Differences between groups were evaluated by either one-way analysis of variance (ANOVA), or the Kruskal–Wallis test, as appropriate. Associations between variables were tested using linear regression analyses (for continuous normally distributed data) or the Spearman rank correlation (for continuous non normally distributed variables). A two-tailed P < 0.05 was considered to indicate statistical significance. The MedCalc ver. 12.0 statistical software package (MedCalc. Software, Mariakerke, Belgium) was used for data analysis.

## Results

**Fig 1:**
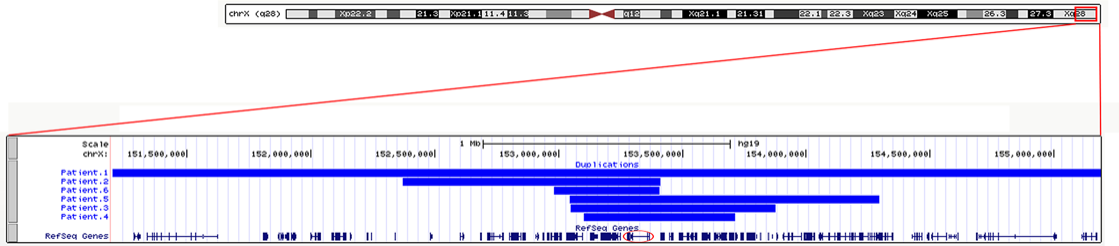
Graphical view of the *MECP2* duplications/triplication. Graphical view of the *MECP2* duplications/triplication was created with custom tracks in the UCSC genome browser (GRCh37/hg19), (Patient 1 was identified with a triplication). The involved regions are shown in blue and *MECP2* is marked by a red circle.

All the enrolled patients showed a *MECP2* duplication/triplication whose position and size was defined according to the GRCh37/hg19 human genome annotation (Fig 1 and Table 1).

**Table 1:** *MECP2* duplication syndrome: clinical features and genetic details.

Clinical features	Patient #1	Patient #2	Patient #3	Patient #4	Patient #5	Patient #6
Age (years)	14	8	2.5	9	13	12
Gender	Male	Female	Male	Male	Male	Male
Microcephaly	Yes (+)	Yes (+)	Yes (+)	Yes (+)	Yes (+)	No
Hand stereotypies	Yes (++)	Yes (+)	Yes (+)	Yes (++)	Yes (+)	No
Abnormal breathing	Yes (+)	Yes (+)	No	Yes (+)	Yes (+)	No
Bruxism	Yes (++)	Yes (+)	Yes (+)	Yes (++)	Yes (+)	No
Laryngomalacia	Yes	No	Yes	No	No	No
Sleep disturbances	Yes (+)	Yes (+)	Yes (+)	Yes (+)	Yes (+)	Yes (+++)
GERD, drooling	Yes (+)	Yes (+)	Yes (+)	Yes (+)	Yes (+)	Yes (+)
Constipation	Yes (++)	Yes (++)	Yes (++)	Yes (+++)	Yes (++)	Yes (++)
Genital abnormalities	No	No	No	Yes (hypospadias)	No	No
Facial dysmorphism	Yes (+)	Yes (+)	Yes (+)	Yes (+)	Yes (+)	No
Facial hypotonia	Yes (+)	Yes (+)	Yes (+)	Yes (+)	Yes (+)	Yes (+)
Dysphagia	Yes (+++)	Yes (++)	No	Yes (+++)	Yes (++)	Yes (+)
Intellectual Disability	Yes (+++)	Yes (+++)	Yes (+++)	Yes (+++)	Yes (+++)	Yes (+++)
Developmental regression	Yes (+)	Yes (+)	Yes (+)	Yes (+)	Yes (+)	Yes (+)
Epilepsy	Yes (+++)	Yes (+++)	No	Yes (+++)	Yes (+++)	Yes (+++)
MRI abnormalities	Yes*	Yes*†¶‡	Yes*¶‡**	Yes**	Yes*	Yes*
Hypotonia/ spasticity	Yes (+++)	Yes (+)	Yes (+)	Yes (+)	Yes (+++)	Yes
Feeding difficulties	Yes (+)	Yes (+)	Yes (+)	Yes (+)	Yes (+)	Yes
Recurrent Infections	Yes^§^	No	Yes^§^	Yes^§^	Yes^§^	Yes
Endocrine abnormalities	No	Yes (hypothyroidism)	No	No	No	No
*MECP2* duplication/triplication• position and size (GRCh37/hg19)	chrX:151198447–155190224• (~4Mb)	chrX:152370000–153410000 (1Mb)	chrX:153049224–153877929 (~828kb)	chrX: 153101077–153713921 (~613kb)	chrX: 153043806–154294453 (~1,2Mb)	chrX: 152982000–153408000 (~426kb)

Yes (+++), Yes (++), and Yes (+) indicated high, medium, and low relevance of the clinical presentation, respectively.

*Central and cortical atrophy

**Periventricular leukomalacia

†immature white matter features, mainly involving frontal lobe

¶Cavum vergae

‡Cyst of the septum pellucidum

^§^recurrent upper and lower respiratory tract infections.

•Patient with *MECP2* triplication, GERD; gastro esophageal reflux disease, MRI; magnetic resonance imaging, Mb; mega base, kb; kilo base.

The typical features of the MDS, including developmental regression, epilepsy, magnetic resonance imaging abnormalities, hypotonia/spasticity were present in all the examined patients with the single exception of epilepsy, reported in five out of six subjects (Table 1).

**Fig 2:**
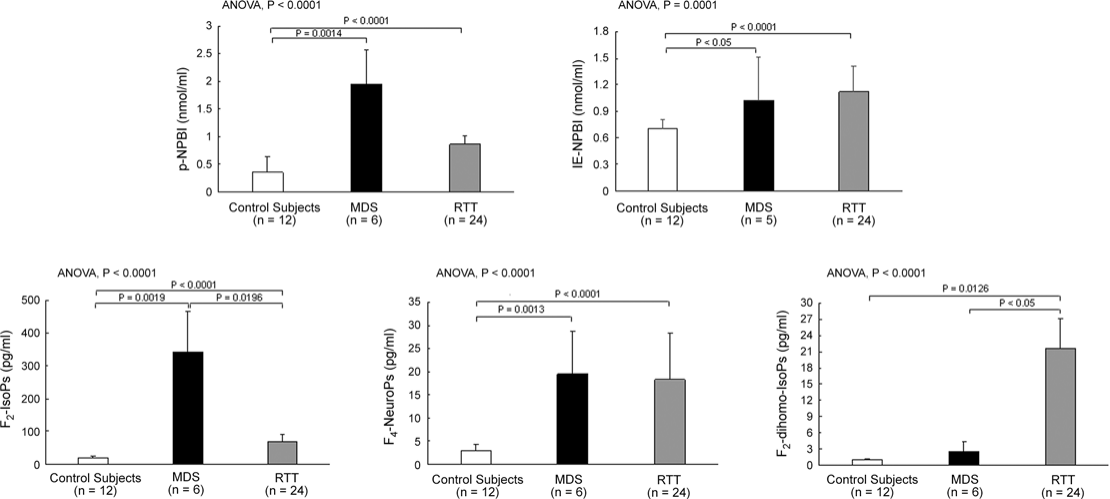
Oxidative stress marker plasma levels in MDS and RTT. Levels of NPBI, plasma free F_2_-IsoPs, F_4_-NeuroPs, and F_2_-dihomo-IsoPs in MDS are compared with those of RTT and healthy control subjects. All the statistical significant differences were reported. Legend: ANOVA, analysis of variance; F_2_-dihomo-isoPs, F_2_-dihomo-isoprostanes; F_2_-IsoPs, F_2_-isoprostanes; F_4_-NeuroPs, F_4_-neuroprostanes; IE-NPBI, intraerythrocyte non protein bound iron; MDS, *MECP2* Duplication Syndrome; p-NPBI, plasma non protein bound; RTT, Rett syndrome.

All the MDS patients showed relevant differences in four out of five of the investigated OS markers, as compared to the healthy controls (Fig 2). In particular, significantly increased plasma levels of p-NPBI and IE-NPBI, forms of redox active iron, and both F_2_-IsoPs and F_4_-NeuroPs, non-enzymatic oxidized products from polyunsaturated fatty acids (i.e., arachidonic and docosahexaenoic acid, respectively), were detected. No significant differences were evidenced for F_2_-dihomo-IsoPs, biomarkers of adrenic acid oxidation. Both examined forms of iron (i.e., p-NPBI and IE-NPBI) are considered pro-oxidant agents [26], whereas the investigated isoprostanoids are indexes of lipid peroxidation (i.e., F_2_-IsoPs) [26], and brain gray (i.e., F_4_-NeuroPs) or white (i.e., F_2_-dihomo-IsoPs) matter oxidative damage [27, 28].

A comparison of the OS status in MDS patients and RTT patients *vs* the healthy control population evidenced similarities, with the exceptions of significantly higher plasma levels of F_2_-IsoPs and lower plasma levels of F_2_-dihomo-IsoPs in MDS. Indeed, the levels of p-NPBI, IE-NPBI, and F_4_-NeuroPs in MDS were found to be comparable to those observed in RTT (Fig 2). Moreover, the levels of all the examined plasma and intraerythrocyte biomarkers were significantly higher in RTT as compared to the control group (Fig 2).

**Fig 3:**
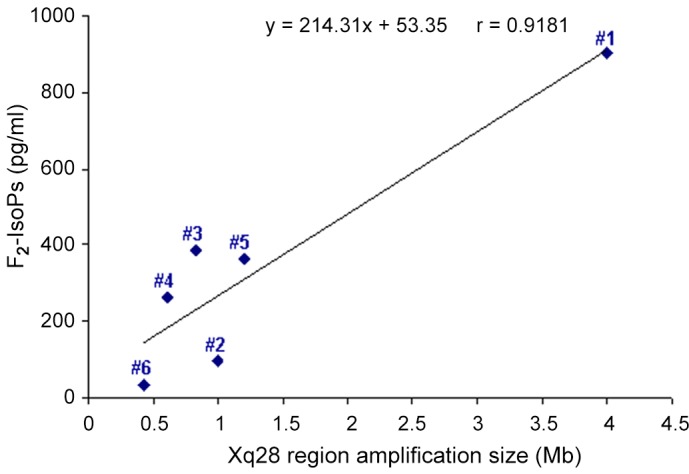
Relationship between plasma F_2_-IsoPs and Xq28 size (univariate regression analysis). A positive linear relationship of plasma F_2_-IsoPs vs. Xq28 duplication/triplication size is showed. The strength of the relationship is indicated by the correlation coefficient (r = 0.9181, P = 0.0098). The linear regression equation was reported.

A significant positive correlation between the Xq28 region duplication/triplication size and plasma levels of F_2_-IsoPs (r = 0.9181; 95% C.I.: 0.4181 to 0.9912; P = 0.0098) was evidenced (Fig 3). Additionally, a correlation trend between the Xq28 region duplication size and the erythrocyte sedimentation rate (ESR) (r = 0.732; P = 0.1598) was observed. On the other hand, no significant correlations were detected between OS markers and age, thus reinforcing the hypothesis of a link between the *MECP2* gain-of-function mutations and OS derangement.

Significant differences were observed for total cholesterol, ESR, and ALC (post-hoc comparison: RTT > MDS ≈ Control Subjects; MDS ≈ RTT > Control Subjects; MDS ≈ RTT > Control Subjects, respectively) (Table 2). In contrast, no significant differences were reported for all the other parameters of the typical clinical-chemical pattern (Table 2). No significant differences were present for immunoglobulin class G and class M, high density lipoproteins-cholesterol, white blood cells, absolute counts for neutrophils, eosinophils, or basophils. (data not shown).

**Table 2:** Routine chemistry biomarkers in patients with *MECP2* duplication syndrome, Rett syndrome, and control subjects.

	*MECP2* duplication syndrome (n = 5)*	Rett syndrome (n = 24)	Control subjects (n = 12)	ANOVA P value
Total cholesterol (mg/dl)	141.8 ± 39.80	172.6 ± 27.3	144.6 ± 16.0	**0.007**
ESR (mm/h)	30.4 ± 21.43	32.68 ± 15.58	9.16 ± 5.85	**<0.001**
ALC (cells x10^3^/mm^3^)	3.32 ± 2.04	3.8 ± 0.85	2.68 ± 0.62	**0.019**
AMC (cells x10^3^/mm^3^)	0.394 ± 0.21	0.737 ± 0.37	0.55 ± 0.15	0.052
Fibrinogen (mg/dl)	332.2 ± 90.8	404.2 ± 92.1	334.7 ± 60.78	0.055
Triglycerides (mg/dl)	89.2 ± 27.29	66.5 ± 15.26	67.8 ± 28.47	0.111
IgA (mg/dl)	201.0 ± 157	117.2 ± 33.4	140.9 ± 34.1	0.101

Values were expressed as means ± standard deviation. The P value are referred to one-way ANOVA tests. Significant P values are highlighted in bold. Non-significant trends for AMC, fibrinogen, triglycerides, and IgA were evidenced.

High density lipoproteins-cholesterol, white blood cells, absolute counts for neutrophils, eosinophils, and basophils were not significant different in a comparison among the three examined populations.

ESR, erythrocyte sedimentation rate; ALC, absolute lymphocytes counts, AMC, absolute monocytes counts, IgA, immunoglobulin class A; mm, millimeter; h, hour.

* data for patient #6 were not available

## Discussion

Enhanced OS has been claimed to be involved in several pathological processes [35], including RTT, a rare genetic disease in which a deficiency of MeCP2 is demonstrated [36, 37]. In particular, we have previously shown that OS in brains of mice with *MECP*2 loss-of-function mutations takes place and can be rescued, along with neurological signs, by *Mecp2* brain specific gene reactivation [19]. However, to date, no hints on the opposite being true exist, *i*.*e*., no evidence is available for supporting the paradoxical concept that “too much” MeCP2 could lead to similar biochemical events as observed in conditions of “too little” MeCP2.

In the present study, we demonstrate, for the first time, the occurrence of a redox imbalance in patients with *MECP2* overexpression. Moreover, our data, while adding new evidence with regard to the *MECP2*-OS link, suggest that a fine tuning of the *MECP2* dosage may play a key role in regulating redox homeostasis in humans.

Whether mitochondria would be the main, or the only major, intracellular sources for the abnormal redox status in MDS and RTT is still to be ascertained [19, 38–41]. To this regard, an ultrastructural analysis of primary cultures of skin fibroblasts from RTT patients has shown no major morphological changes in the mitochondria [42]. At the same time, to the best of our knowledge, no studies on mitochondrial function in MDS have been published to date.

Oxidized products from polyunsaturated fatty acids appear to be promising molecules to be investigated in MDS, as they could mirror an ongoing oxidative brain damage. To this regard, in RTT mouse models, the presence of isoprostanes was evidenced in brain concomitant to elevated isoprostane plasma levels [19]. The reason for the strongly increased F_2_-IsoPs plasma levels in patients presenting *MECP2* gain-of-function mutations remains to be elucidated. Nevertheless, the evidenced dose-effect relationship between Xq28 duplication size and F_2_-IsoPs production further supports the existence of a close link between *MECP2* and redox homeostasis control. A close link between *MECP2* gene expression and F_2_-IsoPs formation has been reported in RTT where the *MECP2* mutations associated to more severe phenotypes exhibited higher F_2_-IsoPs plasma levels [17].

Interestingly, increased F_4_-NeuroP plasma levels in both MDS and RTT (Fig 2), suggest the involvement of the gray matter oxidative damage in both conditions. Currently, F_4_-NeuroPs are investigated as potential biomarker for neurological disease. In human RTT, F_4_-NeuroPs has been related to neurological symptoms severity, mutation type and clinical presentation [27], whereas their levels were significantly elevated in the brain of RTT symptomatic null mice [19]. The relevance of F_4_-NeuroPs in the neurological disease is also reported for the pathogenesis of Alzheimer’s disease [43, 44].

Gain-of-function and loss-of-function *MECP2* mutations were found to generate distinct F_2_-dihomo-IsoPs patterns, oxidized fatty acid products considered as biomarkers of free radical damage to myelin [28, 45]. Although a reduced volume of the brain white matter has been recently reported in patients affected by Xq28 duplication involving the *MECP2* gene [46], our data might suggest a differential involvement of the brain white matter damage for RTT and MDS, on the base of the recovered F_2_-dihomo-IsoPs plasma levels.

This peculiar OS markers pattern further reinforces the specificity of the *MECP2*-OS link, which, far from being an epiphenomenon, appears to be intimately related to the specific *MECP2*-related disorders [17]. For some of those molecules known to be implicated in the general regulation of the redox homeostasis, an epigenetic modulation by MeCP2 has already been demonstrated [20]. To this regard, a possible role for contiguous genes to *MECP2*, *i*.*e*., *IRAK1* (OMIM *300283), cannot be ruled out, as a compensatory upregulation of *IRAK1* has been reported in *Mecp2* loss-of-function mutations associated with experimental RTT murine models [47, 48].

Given that MDS and RTT both lead to severe neurodevelopmental disorders sharing several similar features (Table 3), our data, beyond stressing the critical role of the *MECP2* function for the OS status, shed further light on the relevance of redox homeostasis in the central nervous system integrity.

**Table 3:** Commonalities and differences between *MECP2* duplication syndrome and Rett syndrome.

	*MECP2* duplication syndrome	Rett syndrome
MeCP2	↑	↓
Irak1	↑	↑/(↓ in deletions)
p-NPBI	↑ **	↑ *
F_2_-IsoPs	↑***	↑ **
F_2_-dihomo-IsoPs (supposed white matter involvement)	↔ / ↑*	↑***
F_4_-NeuroPs (supposed gray matter involvement)	↑***	↑***
IE-NPBI	↑•	↑•
Inflammation	Yes	Yes
Gender	Male/Female	Female
Microcephaly	Yes (83.3%)	Yes
Hand stereotypies	Yes (83.3%)	Yes (+++)
Abnormal breathing	Yes (66.6%)	Yes (+++)
Bruxism	Yes (83.3%)	Yes (+++)
Laryngomalacia	Yes (16.6%)	No
Sleep disturbances	Yes (100%)	Yes (+++)
Gastro esophageal reflux disease (GERD), drooling	Yes (100%)	Yes
Constipation	Yes (100%)	Yes
Genital abnormalities	Yes (16.6%)	No
Facial dysmorphism	Yes (83.3%)	No
Facial hypotonia	Yes (100%)	No
Dysphagia	Yes (83.3%)	Yes
Intellectual disability	Yes (100%)	Yes
Developmental regression	Yes (100%)	Yes
Epilepsy	Yes (83.3%)	Yes
Magnetic resonance imaging (MRI) abnormalities	Yes (100%)	Rare
Hypotonia/ spasticity	Yes (100%)	Yes
Feeding difficulties	Yes (100%)	Yes
Recurrent Infections	Yes (66.6%)	Yes/No
Endocrine abnormalities	Yes (16.6%)	Yes/No
Hypoxia	Not evaluated	Yes (mild chronic)

↑, ↓, and ↔ indicate increased, decreased and similar levels, as compared to control subjects, respectively. Yes (+++) indicated high relevance of the clinical presentation, respectively.

•, *, **, ***, indicate <100, 100–250, 250–500, and >500 percentage increase, respectively. p-NPBI, plasma non protein bound; IE-NPBI, intraerythrocyte non protein bound iron; F_2_-IsoPs, F_2_-isoprostanes; F_4_-NeuroPs, F_4_-neuroprostanes; F_2_-dihomo-isoPs, F_2_-dihomo-isoprostanes

Alike RTT, MDS seems to elicit an inflammatory response. This condition could be linked to either imbalanced gene control or abnormal redox status. Although recurrent infections are a quite common feature of MDS [49], present in the clinical history of five out of our six patients, no signs of ongoing infection were detectable at the time of the blood sampling. However, it is also possible that the elevated ESR values are not directly related to the underlying genetic abnormality. Nevertheless, the inflammatory similarities observed in MDS and RTT well comply with the potential role for MeCP2 in regulating cytokine-dependent T helper cell differentiation [20]. Thus, *MECP*2 seems to exert a finely tuned regulation of major biological processes.

## Conclusion

Taken together, our data indicate that a *MECP2* disequilibrium, either due to loss-of-function or gain-of-function mutation, leads to similar phenotypes in terms of OS status, thus adding new evidence on the relationship between OS and *MECP2*.
